# Development and validation of a simplified risk prediction model for preterm birth: a prospective cohort study in rural Ethiopia

**DOI:** 10.1038/s41598-024-55627-z

**Published:** 2024-02-28

**Authors:** Eskeziaw Abebe Kassahun, Seifu Hagos Gebreyesus, Kokeb Tesfamariam, Bilal Shikur Endris, Meselech Assegid Roro, Yalemwork Getnet, Hamid Yimam Hassen, Nele Brusselaers, Samuel Coenen

**Affiliations:** 1 Department of Family Medicine & Population Health, Faculty of Medicine and Health Sciences, University of Antwerp, Antwerp, Belgium; 2 Departmentof of Nutrition and Dietetics, School of Public Health, Addis Ababa University, Addis Ababa, Ethiopia; 3 Department of Food Technology, Safety, and Health, Faculty of Bioscience Engineering, Ghent University, Ghent, Belgium; 4 Department of Reproductive Health and Health Service Management, School of Public Health, Addis Ababa University, Addis Ababa, Ethiopia; 5 Global Health Institute, Department of Family Medicine & Population Health, Antwerp University, Antwerp, Belgium; 6 Centre for Translational Microbiome Research, Department of Microbiology, Tumour and Cell Biology, Karolinska Institute, Stockholm, Sweden; 7 Centre for General Practice, Department of Family Medicine & Population Health, Faculty of Medicine and Health Sciences, University of Antwerp, 2000 Antwerp, Belgium

**Keywords:** Prediction model, Preterm birth, Risk score, Pregnant women, Ethiopia, Medical research, Epidemiology, Preclinical research, Health care, Pregnancy outcome

## Abstract

Preterm birth is one of the most common obstetric complications in low- and middle-income countries, where access to advanced diagnostic tests and imaging is limited. Therefore, we developed and validated a simplified risk prediction tool to predict preterm birth based on easily applicable and routinely collected characteristics of pregnant women in the primary care setting. We used a logistic regression model to develop a model based on the data collected from 481 pregnant women. Model accuracy was evaluated through discrimination (measured by the area under the Receiver Operating Characteristic curve; AUC) and calibration (via calibration graphs and the Hosmer–Lemeshow goodness of fit test). Internal validation was performed using a bootstrapping technique. A simplified risk score was developed, and the cut-off point was determined using the “Youden index” to classify pregnant women into high or low risk for preterm birth. The incidence of preterm birth was 19.5% (95% CI:16.2, 23.3) of pregnancies. The final prediction model incorporated mid-upper arm circumference, gravidity, history of abortion, antenatal care, comorbidity, intimate partner violence, and anemia as predictors of preeclampsia. The AUC of the model was 0.687 (95% CI: 0.62, 0.75). The calibration plot demonstrated a good calibration with a *p*-value of 0.713 for the Hosmer–Lemeshow goodness of fit test. The model can identify pregnant women at high risk of preterm birth. It is applicable in daily clinical practice and could contribute to the improvement of the health of women and newborns in primary care settings with limited resources. Healthcare providers in rural areas could use this prediction model to improve clinical decision-making and reduce obstetrics complications.

## Introduction

Preterm birth, as defined by the World Health Organization (WHO), is birth before 37 completed weeks of gestation and poses a significant global health challenge^1^. A substantial proportion, approximately 65–70% of preterm births, occur spontaneously^2^, and its associated deaths are more common in low and middle-income countries (LMICs) ^3,4^. An estimated 15 million infants are born prematurely each year, constituting a considerable public health concern^5^.

In 2014, 14.8 million (10.6%) live births were preterm, with 80% of cases concentrated in Asia and Sub-Saharan Africa. The rate of preterm birth varies between countries, from 8.7% in Europe to 13.4% in North Africa. India, China, Nigeria, Bangladesh, and Indonesia account for 44.6% (6.6 million) of preterm births worldwide^6^. within Ethiopia alone, 320,000 neonates are born preterm annually, reflecting a prevalence rate of approximately 10.5% ^7,8^.

The burden of preterm birth is a serious public health problem that contributes to significant neonatal morbidity and mortality^9^. Preterm birth is associated with short and long-term morbidities for survivors, incurring high costs for the healthcare system and psychological and financial consequences on the family^10,11^. Preterm birth is one of the most common obstetric complications^12^. In 2016, prematurity was the leading cause of neonatal death during the first weeks of life for children under five years^13^. Approximately 35% of neonatal deaths^14^ and 18% of all deaths of under-five children^4^ were attributable to preterm birth. In Ethiopia, preterm birth complications account for 10% of all deaths of children under five years^7^. Maternal age less than 20 years^15^, short stature (≤ 155 cm)^16^, smoking^17^, anemia, malaria infection^18^, intimate partner violence^19^, multiple pregnancies, pre-existing chronic conditions^20^, rural residence, short birth interval, history of abortion^21^, history of preterm birth^8^ and household food insecurity^22^ are the potential risk factors of preterm birth. Stress is also a widespread psychological health problem among pregnant women, and it contributes to preterm birth^23,24^.

Despite preterm birth being a global public health priority, success in reducing adverse outcomes through evidence-based policies during antenatal care has been limited^9,20^. Early identification and quantification of individual women at risk of preterm birth could help to improve the quality of care during pregnancy, ensuring that all women have a positive pregnancy experience and outcomes^5^. Predicting preterm birth would allow earlier intervention to reduce infant morbidity and mortality, benefiting families, society, and healthcare.

The potential preterm birth risk factors have been identified, but the predictive value of their combination remains unclear in rural settings. Prediction models are vital for healthcare providers to estimate probabilities of preterm birth and allow for timely intervention to reduce adverse outcomes^25^. Ultrasound examinations^26,27^ and biomarker tests^28,29^ have been utilized to predict preterm birth, but these methods are less practical in resource-limited settings due to a lack of specialized medical equipment and trained health care providers^30,31^. Furthermore, existing studies on the prediction of preterm birth often exhibit limitations, such as lack of inclusivity of important prognostic factors^32–34^, focused on hospitalized women ^35–38^ or exclusively on multiple pregnancies^39,40^ and reliance on high-level health care settings^33,34^. However, none of these studies were accurate enough to be applied in daily clinical practice in the primary care setting due to disparities in the potential predictors, domains, and levels of healthcare settings. We, therefore, set out to develop a simple prediction tool to identify pregnant women at higher risk of preterm birth in early pregnancy in resource-limited primary care settings.

The current study aimed to develop and validate a simplified risk prediction model for preterm birth and evaluate the added value of maternal stress in predicting preterm birth using the routine characteristics of pregnant women in the rural area of Ethiopia.

## Methods

### Study setting and design

We used data from the Butajira Nutrition, Mental Health, and Pregnancy (BUNMAP) project among pregnant women and their newborns in Southern Ethiopia. The BUNMAP project was established in 2016 under the Butajira Health and Demographic Surveillance Site (BHDSS), consisting of nine rural and one urban dweller association. The BHDSS is one of the oldest Demographic and Health Survey sites in the Southern Ethiopia region^41^.

The BUNMAP project was an open, prospective cohort of pregnant women and their offspring (up to 59 months) collected between 2017 and 2019. We developed a prediction model for preterm birth using the baseline characteristics of pregnant women and birth history. The theoretical design was the incidence of preterm birth as a function of multiple predictors of ambulatory pregnant women. Health extension workers in the BHDSS identified pregnant women aged 15 to 49 through house-to-house surveillance.

### Study population

**Figure 1:**
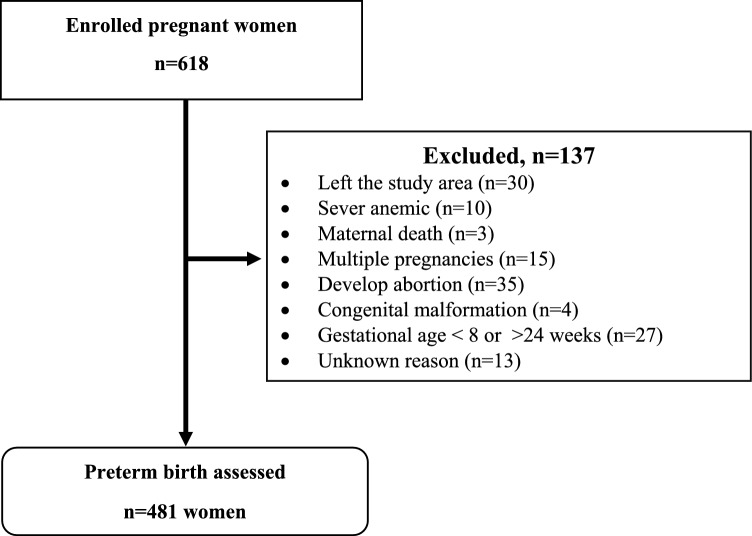
Flow chart of the study design for preterm births in the Butajira Nutrition, Mental Health, and Pregnancy cohort.

At baseline, all pregnant women aged 15–49 years with gestational age between 8 and 24 completed weeks, living in the Butajira HDSS, and planning to deliver in the study area were included in the study. Pregnant women who were severely malnourished or had a mid-upper arm circumference (MUAC) of less than 17.5 cm and severely anemic women with hemoglobin (Hgb) levels less than seven g/dL were excluded from the cohort. Our study included pregnant women enrolled in the cohort who met the inclusion criteria and had a well-determined gestational age at birth (Fig. 1**)**.

### Predictors and outcome assessment

After enrolment, all women were requested to travel to the nearest health facility for a comprehensive baseline assessment.

An experienced sonographer used transabdominal, portable diagnostic imaging, and full-color flow mapping ultrasound to confirm gestational age at baseline. The primary outcome, preterm birth, was defined as birth before 37 completed weeks of gestation. The study questionnaire was adopted and developed from the validated tools and Ethiopian demography and health survey to collect data on the following predictors: Intimate partner violence (IPV), maternal stress, maternal age, marital status, educational status, pregnancy type, substance use, Mid-upper arm circumference (MUAC), Comorbidity, history of previous and current Antenatal care (ANC) visits, history of abortion, history of preterm birth, gravidity, contraceptive use, deworming, and comorbidity. Then, potential predictors were categorized by considering the clinical relevance thresholds for adverse birth outcomes.

*Mid-upper arm circumference* was measured using a standard MUAC tape, and less than 23 cm was considered maternal malnutrition^42^.

*Intimate partner violence* was assessed using the Hurt, Insult, Threaten, and Scream (HITS) screening tool. This four-item questionnaire asks respondents how frequently their partner physically hurt, insulted, threatened harm, and screamed at them during pregnancy using a five-point Likert scale ranging from "never" (coded as 1) to "frequently" (coded as 5). The total score ranges from 4 to a maximum of 20. Positive or exposure to IPV was considered if the HITS score was more significant than ten^43^.

*Maternal stress* was assessed based on the ten-item classic perceived stress scale (PSS) assessment instrument for one month before data collection day using a five-point Likert scale ranging from "never" (coded as 0) to "frequently" (coded as 4). The individual score on the PSS can range from 0 to a maximum of 40. Exposure to low, moderate, or high maternal stress was considered if the PSS score was 0–13, 14–26, or 27–40, respectively^44^.

*Maternal anemia* was assessed by measuring Hgb concentration in red blood cells by taking a finger-prick blood sample using a Hemo-Cue (Hb-201) instrument. Pregnant women with Hgb concentration < 11 g/dl were considered anemic^45^^.^

*Maternal comorbidity* was considered when one or more of the following medical conditions exist: cardiac disease, diabetes, thyroid disease, chronic hypertension, HIV infection, malaria, typhoid, or renal disease in the baseline assessment^46^.

*Substance use* was examined in pregnant women who consumed local alcohol or beer or chewed khat at least once a week during pregnancy.

### Statistical analysis

The data were collected using the Open Data Kit (ODK) platforms and were exported to the R statistical programming software version 4.2.0^47^. The baseline characteristics of the women were summarised in a table with frequencies and proportions. The distribution was assessed using histograms for maternal age, gestational age, and MUAC at baseline. The median and interquartile range (IQR) for the pregnant women’s age, gestational age, and MUAC were presented. The mean and standard deviation (SD) were also used to present the baseline Hgb level of women. We performed Little’s missing completely at random (MCAR) test and checked the pattern of missing values. The p-value (< 0.001) indicated that the missing was not MCAR. However, the test result is insufficient to indicate whether the missing is not missing at random (NMAR) or missing at random (MAR)^48^. We then performed multiple imputations by chained equation using the “mice” package with ten imputations and 20 iterations^49^. Sensitivity analyses were performed to determine whether the MAR assumption was valid. The MAR assumption was valid, and the complete case and imputed data analysis results were comparable (Supplementary Table S1).

#### Model development

Individual predictors that significantly contribute to the risk of preterm birth were examined using univariable logistic regression analysis. The Likelihood Ratio Test (LRT) was used to determine the p-value for each model. Variables with a p-value for the LRT less than 0.25 were considered eligible to be included in the multivariable logistic regression analysis. The backward stepwise elimination technique with a p-value ≤ 0.15 for the LRT was fitted to build a final multivariable logistic regression model. The predictive accuracy of the final model was checked using discrimination (AUC) and calibration (calibration graphs and Hosmer–Lemeshow goodness of fit test) parameters. The Hosmer–Lemeshow goodness of fit test with a p-value greater than 0.05 indicates good calibration, which means that the probability of preterm birth estimated by the model is similar to the observed probability. An AUC of 0.5 indicates no discrimination ability, while an AUC of 1 indicates perfect discrimination.

Moreover, the added value of maternal stress in predicting preterm birth was assessed using logistic regression analysis. The model’s performance, including maternal stress, was evaluated using discrimination and calibration. The AUC of this model was compared with the reduced model to evaluate the improvement in prediction performance.

#### Internal validation

The model was internally validated using a bootstrap technique^50^ to estimate the degree of over-optimism of the final model when applied to a similar population. Internal validation was performed on the regression coefficient with a 95% confidence interval (CI) and the AUC of the model using 2,000 random bootstrap samples. The AUC difference between the bootstrap and the original full sample measured the optimism of the predictive model.

### Simplified risk score development

Based on the final model's regression coefficients hierarchy, a simplified risk score was computed to provide an easily applicable prediction model. Each regression coefficient of the predictors in the final model was divided by the smallest regression coefficient, and the result was rounded to the nearest integers. The risk score performance was assessed and compared with the original regression coefficient model using the AUC. The simplified risk score is also arbitrarily classified based on the size of each interval and its potential public health relevance.

The TRIPOD (transparent reporting of a multivariable prediction model for individual prognosis or diagnosis) checklist^51^ was used to guide the development and validation of the prediction model and reporting (Supplementary Table S2).

### Ethics declarations

The study has been reviewed, and ethical clearance was obtained from the Institutional Review Boards of Addis Ababa University, College of Health Sciences (code: 099/17/SPH). Written informed consent and parental assent were obtained from study participants. This manuscript was approved by the Faculty of Medicine and Health Sciences, University of Antwerp, Belgium, and all methods were carried out in accordance with relevant guidelines and regulations.

## Result

### Characteristics of pregnant women

Of the 618 pregnant women, 137 were excluded from the analysis due to absence or inappropriate determination of gestational age at birth. At enrolment, the median age was 26 with an IQR of 6 years, and 38 (8.1%) were younger than 20 years (Table 1). All women (100%) were married, and 205 (42.7%) did not receive formal education.

**Table 1:** Baseline sociodemographic, obstetric, and clinical characteristics of study participants (*n* = 481).

Category	Missing (%)	Preterm birth		Frequency (%)
		Yes (%)	No (%)	
Age	11 (2.3)			
< 20		4 (10.5)	34 (89.5)	38 (8.1)
20–34		81 (19.8)	327 (80.1)	408 (86.8)
≥ 35		5 (20.8)	19 (79.2)	24 (5.1)
Educational status	1 (0.2)			
No formal education		38 (18.5)	167 (81.4)	205 (42.7)
Primary school		42 (18.9)	180 (81.1)	222 (46.3)
Secondary and above		14 (26.4)	39 (73.6)	53 (11.0)
MUAC	0 (0.0)			
< 23 cm		23 (26.7)	63 (73.3)	86 (17.9)
≥ 23 cm		71 (18.0)	324 (82.0)	395 (82.1)
Anemia	24 (5.0)			
Yes		12 (40.0)	18 (60.0)	30 (6.6)
No		79 (18.5)	348 (81.5)	427 (96.6)
IPV	3 (0.6)			
Negative		78 (18.1)	353 (81.9)	431 (90.2)
Positive		16 (34.0)	31 (66.0)	47 (9.8)
Type of pregnancy	2 (0.4)			
Planned		19 (22.9)	64 (77.1)	396 (82.7)
Unplanned		75 (18.9)	321 (81.1)	83 (17.3)
Comorbidity^#^	9 (1.9)			
Yes		13 (36.1)	23 (63.9)	36 (7.6)
No		76 (17.4)	360 (82.6)	436 (92.4)
Substance use^¶^	6 (1.3)			
Yes		9 (15.0)	51 (85.0)	60 (12.6)
No		85 (20.5)	330 (79.5)	415 (87.4)
Gravidity	77(16.0)			
Primigravida		34 (26.0)	97 (74.0)	131 (32.4)
Multigravida		51 (18.7)	222 (81.)	273 (67.6)
History of abortion	7 (1.5)			
Yes		10 (43.5)	13 (56.5)	23 (4.9)
No		82 (18.2)	369 (81.8)	451 (95.1)
History of preterm	7 (1.5)			
Yes		2 (33.3)	4 (66.7)	6 (1.3)
No		90 (19.2)	378(80.8)	468 (98.7)
History of contraceptive use	2 (0.4)			
Yes		22 (18.5)	97 (81.5)	119 (24.8)
No		72 (20.0)	288 (80.0)	360 (75.2)
History of ANC follow-up for the previous pregnancy	37 (7.7)			
No		15 (18.1)	68 (81.9)	83 (18.7)
Yes		73 (20.2)	288 (79.8)	361 (81.3)
History of ANC for the current pregnancy	2 (0.4)			
Yes		80 (18.4)	355 (81.6)	435 (90.8)
No		14 (31.8)	30 (68.2)	44 (9.2)
Deworming	54(11.2)			
No		5 (14.7)	29 (85.3)	393 (92.0)
Yes		79 (20.1)	314 (79.9)	34 (8.0)
Maternal stress	2 (0.4)			
Very low stress		15 (12.8)	102 (87.2)	117 (24.4)
Moderate stress		73 (21.1)	273 (78.9)	346 (72.2)
High stress		6 (37.5)	10 (62.5)	16 (3.3)

^**#**^Comorbidity considered when one or more of the following medical conditions exist: cardiac disease, diabetes, thyroid disease, chronic hypertension, HIV infection, malaria, typhoid, or renal disease; ^¶^Substance use: exposed to either local alcohol or beer or chewing khat at least once per week; MUAC, Mid-Upper Arm Circumference; ANC, antenatal care, IPV, intimate partner violence.

### Prediction model

Preterm birth occurred in 94 (19.5%) of the 481 women who gave birth in the BHDSS. The multivariable logistic regression analysis included variables with a p-value of less than 0.25 for the LRT in the univariable logistic regression analysis, such as MUAC, gravidity, history of abortion, anemia, comorbidity, IPV, and history of ANC for the current pregnancy. The final prediction model combined seven predictors, including MUAC, gravidity, history of abortion, comorbidity, IPV, anemia, and history of ANC for the current pregnancy (Table 2).

**Table 2:** Association between predictors and preterm birth in South Ethiopia (*n* = 481).

Variables	Univariable analysis	P-value	Adjusted analysis	P-value
	OR (95%CI)		OR (95%CI)	
Age				
< 20	0.45 (0.16, 1.32)	0.278	–	
≥ 35	1.03 (0.37, 2.84)		–	
20–34	Ref		Ref	
Educational status				
No formal education	0.64 (0.31, 1.29)	0.431	–	
Primary	0.65 (0.32, 1.32)		–	
Secondary or above	Ref		Ref	
Mid-upper arm circumference				
< 23 cm	1.67 (0.97, 2.87)	0.072*	1.50 (0.99, 4.89)	0.154**
≥ 23 cm	Ref		Ref	
Gravidity				
Primigravida	1.47 (0.87, 2.49)	0.13	1.77 (1.06, 2.96)	0.033**
Multigravida	Ref		Ref	
History of abortion				
Yes	3.26 (1.38, 7.68)	0.01*	2.73 (1.04, 7.18)	0.051**
No	Ref		Ref	
History of preterm				
Yes	3.26 (1.38, 8.98)	0.467	–	
No	Ref		–	
Anemia				
Yes	2.75 (1.26,6.01)	0.015*	2.15 (0.93, 4.94)	0.083**
No	Ref		Ref	
Intimate partner violence				
Positive	2.33 (1.22, 4.47)	0.014*	2.26 (1.12, 4.55)	0.025**
Negative	Ref		Ref	
Contraceptive use				
Yes	0.91 (0.53, 1.54)	0.721	–	
No	Ref		Ref	
Type of pregnancy				
Unplanned	1.27 (0.72, 2.24)	0.412	–	
Planned	Ref		Ref	
History of ANC follow-up				
No	0.95 (0.50, 1.80)	0.678	–	
Yes	Ref		Ref	
ANC follow-up on current pregnancy				
No	2.08 (1.05, 4.09)	0.043*	2.14 (1.03, 4.42)	0.049**
Yes	Ref		Ref	
Deworming				
Yes	0.80 (0.34, 1.91)	0.555	–	
No	Ref		Ref	
Comorbidity^#^				
Yes	2.78 (1.36, 5.70)	0.013*	2.20 (0.99, 4.89)	0.062**
No	Ref		Ref	
Substance use				
Yes	0.68 (0.32, 1.43)	0.273	–	
No	Ref		Ref	

*p-value of LRT < 0.25 and included in multivariable analysis **p-value of LRT < 0.15 and fitted in the final model such as MUAC, gravidity, intimate partner violence, anemia, history of abortion, history of ANC, and comorbidity: ^**#**^Comorbidity considered when one or more of the following medical conditions exist: cardiac disease, diabetes, thyroid disease, chronic hypertension, HIV infection, malaria, typhoid, or renal disease; (–) not included in multivariable analysis (p-value > 0.25); ANC: Antenatal Care; MUAC: Mid-Upper Arm Circumference; Ref: Reference.

### Model performance and validation

**Figure 2:**
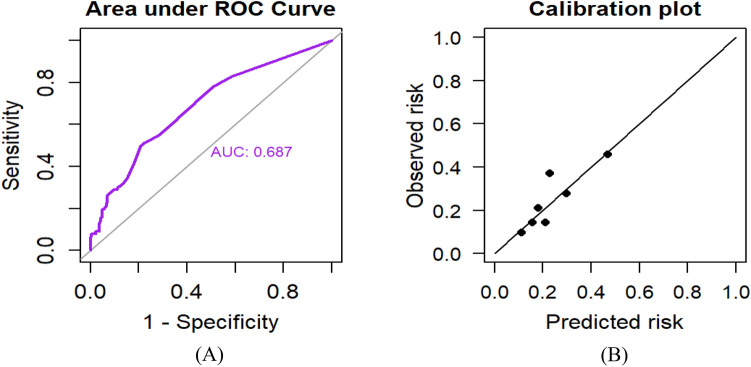
Model performance of the prediction model for preterm birth. (**A**) The area under the receiver operating characteristics curve to evaluate the discrimination. The AUC suggests that the model has a 68.7% (95%CI:62.0%,75.3%) chance to correctly distinguish a high risk for preterm birth from normal pregnancy based on the characteristics of pregnant women in resource limited setting. (**B**) Calibration plot to evaluate the calibration of the prediction model. The visual calibration plot between the observed and prediction risk in different percentiles of the predicted values. The p-value of the calibration plot is 0.713.

The AUC of the final model was 0.687 (95%CI: 0.620, 0.753). The calibration test of the model had a p-value of 0.7134, indicating good agreement between the predicted and observed probability of preterm birth (Fig. 2**)**.

**Figure 3:**
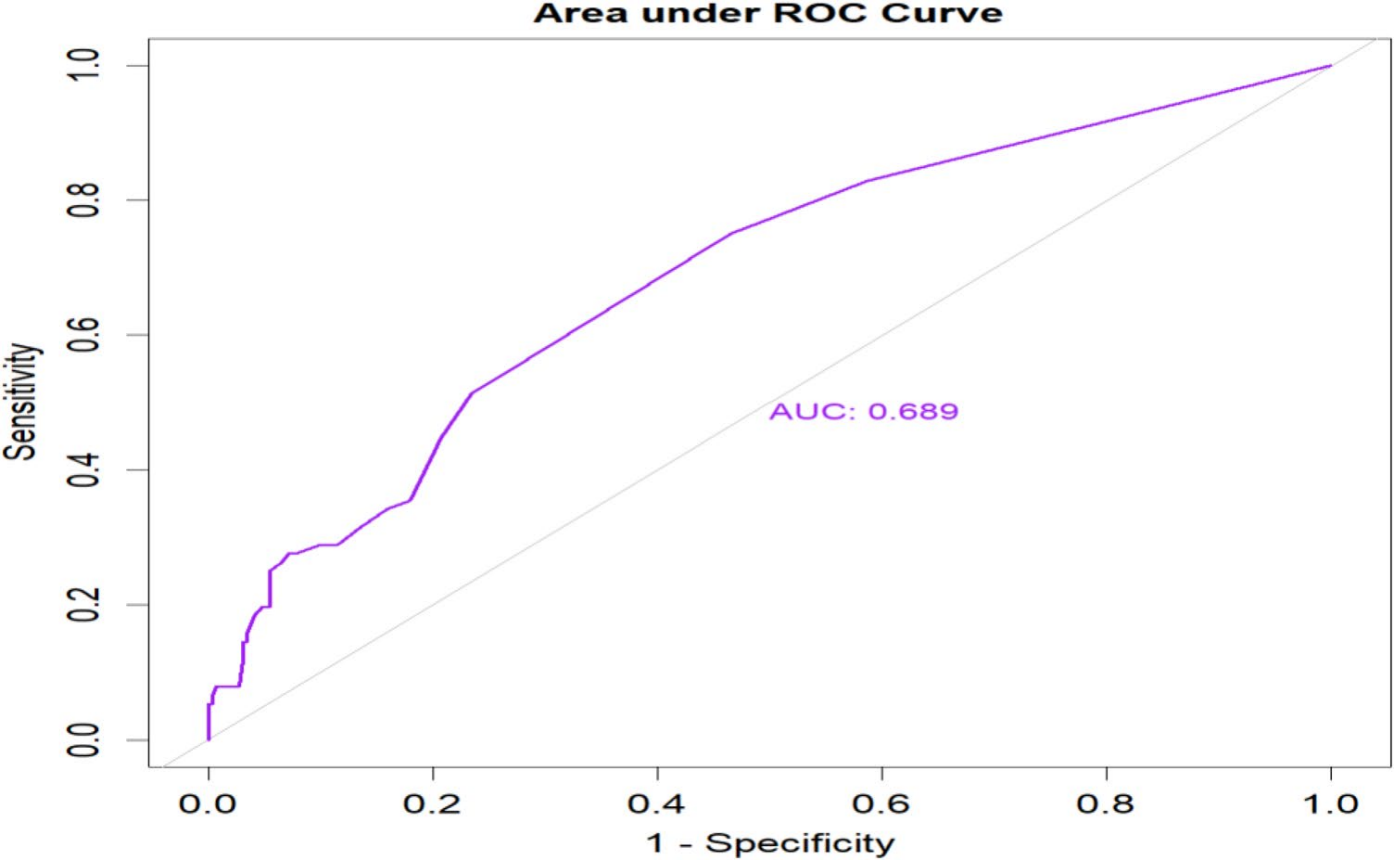
Area under the receiver operating characteristic curve of an internal validation of the prediction model.

The bootstrapping technique showed that we expect low optimism when applied to newly pregnant women in a similar population. The adjusted AUC of the model was 0.689 (95% CI: 0.622, 0.755) with an AUC overoptimism coefficient of 0.002 (p-value = 0.796) (Fig. 3).

### Clinical importance of maternal stress

**Figure 4:**
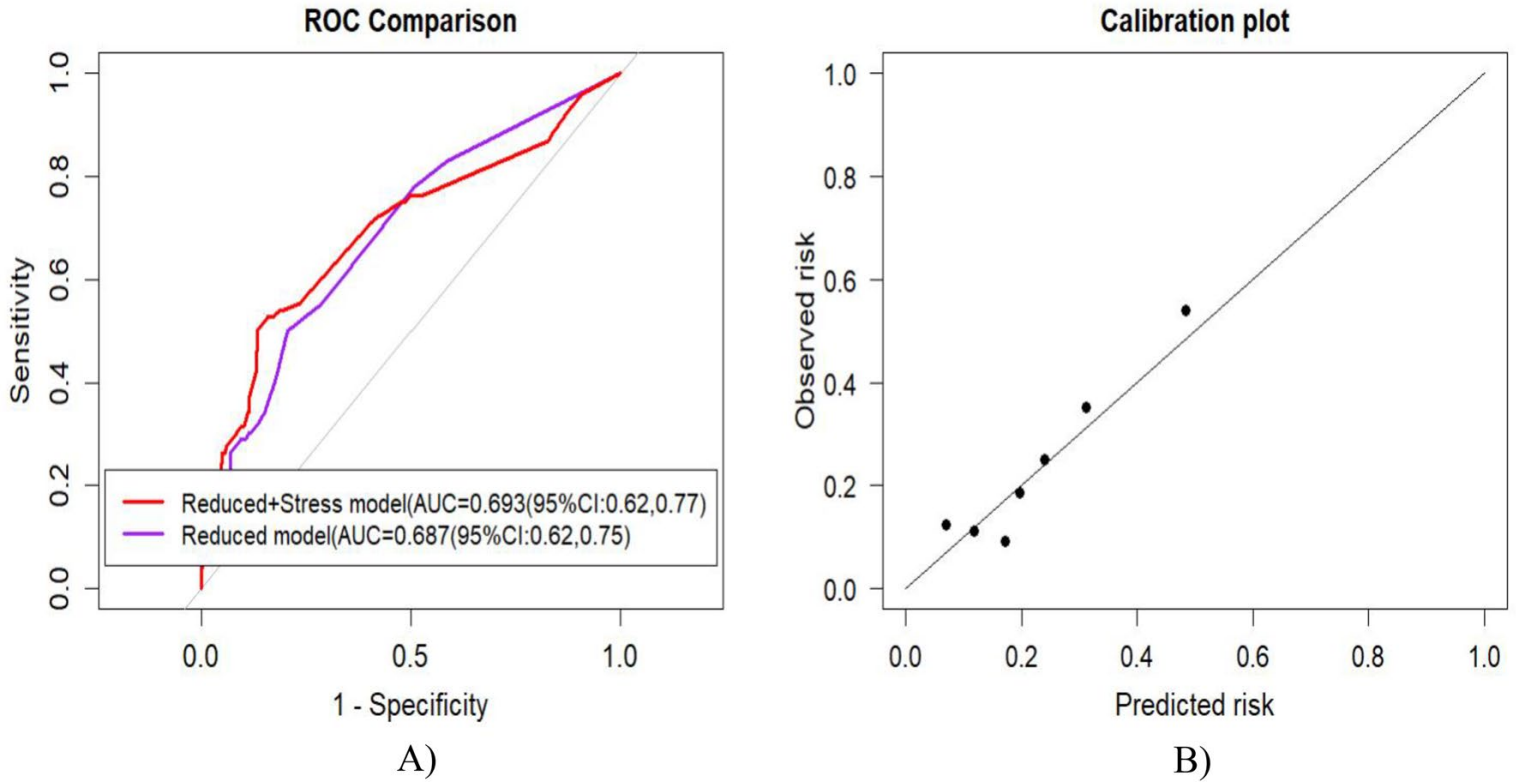
Model performance after adding maternal stress in the prediction model. (**A**) Area under the receiver operating characteristic curve of the final model with and without maternal stress. (**B**) Calibration plot to evaluate the calibration of the addition of maternal stress to the final model. The added value maternal stress was assessed through a calibration plot, revealing a good calibration (a p-value of 0.825).

In the univariable and multivariable analysis, the maternal stress had a p-value for the LRT of 0.031 and 0.008, respectively. The calibration plot indicates that the model, including maternal stress, had good calibration (p-value = 0.825) (Fig. 4). The AUC increased from 0.687 (95%CI: 0.620, 0.753) to 0.693 (95% CI: 0.620–0.766) with a p-value of 0.784. Therefore, the addition of maternal stress to the prediction model has not made a significant difference in the discrimination performance of the model.

### Simplified risk score per individual

A MUAC less than 23 cm had the smallest regression coefficient and was weighted as 1. The total risk score ranged from 0 to 13. The simplified risk score had an AUC of 0.678 (95% CI: 0.612–0.743) and a p-value of 0.08 compared to the original regression coefficient model. Total risk score formula = (1*MUAC < 23 cm + 1*primigravida + 2*being anemic + 2*positive IPV + 3*had a history of abortion + 2* had no ANC history for the current pregnancy + 2*presence of comorbidity) (Table 3).

**Table 3:** The simplified risk score and rounded weight to predict preterm birth.

Variables	Coefficient*	Rounded weight**
MUAC < 23 cm	0.403	1
Primigravida	0.570	1
Anemic	0.766	2
Positive intimate partner violence	0.816	2
Had a history of abortion	1.01	3
Had no History of ANC for the current pregnancy	0.760	2
Comorbidity^**#**^	0.787	2
Total weight ^¶^		13

*Regression coefficient of each predictor; **the weight of each regression coefficient divided by 0.403 and rounded to the nearest integers; ^**#**^Comorbidity was considered when one or more of the following medical conditions exist: cardiac disease, diabetes, thyroid disease, chronic hypertension, HIV infection, malaria, typhoid, or renal disease; ANC: Antenatal Care; MUAC: Mid-Upper Arm Circumference; ^**¶**^total weight: the total rounded risk score of the predictors. The probability of preterm birth = 1/ {1 + exp−(−2.10 + 0.403*MUAC < 23 cm + 0.570*primigravida + 0.766*being anemic + 0.816*positive for intimate partner violence + 1.01*had a history of abortion + 0.760*had no a history of ANC for the current pregnancy + 0.787*presence of comorbidity)].

Table 4 indicates that the risk of preterm birth increased as the simplified risk score increased. The proportion of preterm births was 244 (13.9%) with a simplified risk score of ≤ 1 and 18 (50%) with a simplified risk score of ≥ 5.

**Table 4:** Risk of preterm birth per individual risk score to predict high risk for preterm birth.

Risk score	Number of women	Number of preterm births	Risk of preterm birth (%)^a^
0–1	244	34	13.9
2–4	109	33	30.7
5–13	18	9	50.0

^a^The risk of preterm birth per individual risk score was calculated by listing the assigned total weight and counting the number of women in each leveled weight

Table 5 presents results from the likelihood ratio of each risk score interval. The likelihood ratio increased as the simplified risk score for the prediction model increased. The simplified risk score between 5 and 13 increased the probability of having a preterm birth in pregnant women by 3.8 times compared to those who did not have a preterm birth.

**Table 5:** Interval likelihood ratio of risk score intervals for the prediction model.

Risk score	Preterm birth		Interval likelihood ratio
	Yes (%)	No (%)	
5–13	11.9	3.1	3.8
2–4	43.4	25.8	1.68
0–1	44.7	71.2	0.63
	100	100	

Based on Youden’s index^52^, the optimal cut-off point was a probability of ≥ 0.221 with a sensitivity of 41% (95% CI: 30, 53), a specificity of 82% (95%CI: 77, 86), and a positive predictive value (PPV) of 36% (95%CI: 26, 48), and a negative predictive value (NPV) of 84% (95%CI: 80, 88). The positive likelihood ratio (LR+) was 2.24 (1.56, 3.21), and the negative likelihood ratio (LR−) was 0.72 (95% CI: 0.60, 0.88). Similarly, the optimal cut-off for the simplified risk score was ≥ 2 using Youden's index. One hundred twenty-seven (34.2%) pregnant women were classified as high risk for preterm birth, while 244 (65.8%) were classified as low risk, with a sensitivity of 55% and specificity of 71% **(**Table 6**)**.

**Table 6:** Performance of simplified risk sore at different cut-off points to predict preterm birth.

Cutoff^a^	Sensitivity (95% CI)	Specificity (95% CI)	PPV (95% CI)	NPV (95% CI)	LR + (95% CI)	LR− (95% CI)	Youden index
≥ 1	0.83 (0.73, 0.91)	0.41 (0.36, 0.47)	0.27 (0.21, 0.33)	0.90 (0.84, 0.95)	1.41 (1.23, 1.63)	0.41 (0.25, 0.69)	0.24
≥ 2	0.55 (0.43, 0.67)	0.71 (0.66, 0.76)	0.33 (0.25, 0.42)	0.86 (0.81, 0.90)	1.92 (1.46, 2.51)	0.63 (0.48, 0.82)	0.26
≥ 3	0.34 (0.24, 0.46)	0.85 (0.80, 0.89)	0.37 (0.25, 0.49)	0.83 (0.79, 0.87)	2.24 (1.49, 3.39)	0.78 (0.66, 0.92)	0.19
≥ 4	0.20 (0.11, 0.30)	0.95 (0.91, 0.97)	0.48 (0.30, 0.67)	0.82 (0.78,0.86)	3.64 (1.89, 7.02)	0.85 (0.76, 0.85)	0.15
≥ 5	0.12 (0.04, 0.23)	0.99 (0.97, 1.00)	0.75 (0.35, 0.97)	0.85 (0.80, 0.89)	15.1 (3.14, 72.83)	0.89 (0.81, 0.98)	0.11
≥ 6	0.08 (0.03, 0.16)	0.99 (0.98, 1.00)	0.75 (0.35, 0.97)	0.81 (0.76, 0.85)	11.6 (2.4, 56.6)	0.93 (0.87, 0.99)	0.07

PPV, positive predictive value; NPV, Negative predictive value; LR +, positive likelihood ratio; LR−, negative likelihood ratio.

^a^Simplified risk score cut-off points.

## Discussion

In the present study, one-fifth of the babies were born prematurely. We developed a prediction model for preterm birth based on the baseline characteristics of pregnant women in rural settings. The model combined MUAC, gravidity, comorbidity, ANC follow-up, history of abortion, IPV, and anemia to predict preterm birth. We developed a simplified risk score to improve the practical applicability of the prediction model in clinical practice in primary healthcare settings without requiring advanced diagnostic tests and imaging.

Previous studies developed a model for predicting preterm birth during pregnancy using ultrasound examination^26,27^ and biomarker tests^28,29^. However, these procedures are limited by their expense and complexity, requiring trained healthcare professionals and specialized equipment. In LMICs, such as Ethiopia, most primary care settings lack access to these advanced laboratory and imaging procedures. Consequently, estimating the risk of preterm birth in resource-limited settings using these methods is challenging. Hence, we developed a model using easily obtained and routinely collected maternal characteristics applicable in primary care settings in Ethiopia.

Prediction models were developed for women with signs of preterm birth in tertiary care centers^35,36,38^. The potential predictors of preterm birth in hospitalized women with preterm birth symptoms may differ from those in non-hospitalized women. The healthcare system in tertiary healthcare is different, and the diagnostic procedures are more advanced and expensive. However, the current study estimated the risk of preterm birth using available characteristics of women in the primary care setting.

Prediction models for preterm birth were initially developed in comprehensive specialized hospitals in northern Ethiopia, utilizing a retrospective study design^33,34^. These studies, however, overlooked fundamental predictors recommended by WHO, such as IPV, Substance use, MUAC, history of contraceptive use, deworming, and maternal stress. The exclusive use of a specialized hospital may have limited the generalizability of findings, potentially reflecting specific patient characteristics or a more selective group seeking specialized care. In contrast, our prediction model was deployed in southern Ethiopia using a prospective study design considering the basic WHO recommendations for potential preterm birth predictors. This was crucial in minimizing recall bias and strengthening model accuracy. Additionally, our research was conducted in a rural community setting, where diagnostic methods and healthcare professionals are more limited compared to specialized hospitals. Consequently, the previously developed model may not be applicable for predicting preterm birth in primary care settings.

Schaaf et al.^53^ predicted the risk of preterm birth with poor calibration and an AUC of 0.63 using a combined 13 potential predictors of preterm birth, including fetal sex and vaginal bleeding before 20 weeks of gestation. However, confirmation of fetal sex requires ultrasound examination, and vaginal bleeding before 20 weeks of pregnancy necessitates advanced diagnostic procedures. Therefore, the study is less applicable in clinical settings with scarce resources. Huang et al.^32^ also developed a prediction model for preterm birth by combining stress and metabolic predictors. Maternal stress biomarkers (cortisol) and metabolites were measured in the serum samples to predict preterm birth. The prediction model yielded an optimum AUC value of 0.895. The current study assessed maternal stress using a perceived stress scale. The addition of maternal stress in the prediction model increases the AUC from 0.687 to 0.693, but the difference was insignificant compared to the original model.

Schaaf et al.^53^ calculated the predictive model at two arbitrary cut-off points of the predictive probability. The incidence of preterm birth has occurred in 3.8% of pregnancies. At a predictive probability of 0.1, the sensitivity was 4.2%, and the specificity was 99.3%. At this cut-off point, the PPV was 19.4%, and the NPV was 96.3%. While in our study, we developed different cut-off points using the Youden index. The optimal predicted probability cut-off point was 0.221, with a sensitivity of 41%, a specificity of 82%, a PPV of 36%, and an NPV of 84%. The LR + value was 2.24, and the LR− value was 0.72. The simplified risk score is highly applicable and easier to use in daily clinical practice.

Our study suggests that a prediction model using the characteristics of pregnant women might be useful in identifying pregnant women at high risk of preterm birth in resource-limited settings. Women categorized as high-risk could be referred for further assessment and therapeutic intervention to prevent preterm birth. A simplified risk score would help produce relevant information for the community, policymakers, and clinical interventions to reduce neonatal morbidity and mortality rates.

The current study included highly applicable and routinely collected characteristics of pregnant women in LMICs. This can be used in daily clinical practice in primary care settings to identify high-risk pregnant women. We further used an internal validation based on a bootstrapping technique to provide unbiased estimates of the high risk for preterm birth. We developed different optimal cut-off points based on the coefficient and risk score, which helps to choose various cut-off points depending on the program goal and the availability of resources.

Our study is subject to limitations. The missingness data, mainly the history of abortion, preterm birth, and ANC for the previous pregnancy, had high missing values. However, we tried to minimize the risk of bias by using multiple imputations to develop the estimated regression coefficient of the prediction model.

When pregnant women experience risk or complication during their pregnancy, healthcare providers may intervene to induce preterm birth, affecting the prediction model and implying a computing risk. Future researchers should consider such issues using advanced analysis techniques. Potential predictors of preterm birth and complications might be different in the first and second trimesters. Developing a prediction model in each trimester thus improves the model's practical applicability and predictive capacity. Future research should consider conducting an extensive and multicentre study to improve the prediction model's generalization and external validation.

## Conclusions

In this study, we developed a prediction model and a score for preterm birth risk stratification in rural Ethiopia. The model can identify pregnant women at high risk of preterm birth. Prediction of high-risk women based on individual characteristics could help to strengthen the clinical decision-making of the primary health care providers to reduce obstetric complications. It would also help produce relevant information for the community, policymakers, and clinical interventions on preventing and treating preterm birth and reducing neonatal morbidity and mortality rates.

### Supplementary Information

Supplementary Tables.

## Data Availability

Data is available from the corresponding author at a reasonable request.

## References

[1] World Health Organization (WHO) (1977). Recommended definitions, terminology, and format for statistical tables related to the perinatal period and use of a new certificate for the cause of perinatal deaths. Modifications recommended by FIGO as amended October 14, 1976. Acta Obstetr. Gynecol. Scand..

[2] Howson, C., Kinney, M. & Lawn. J. March of dimes, PMNCH, save the children, WHO. *Born Too Soon: The Global Action Report on Preterm Birth* (World Health Organization, 2012).

[3] World Health Organization(WHO). *Born Too Soon: The Global Action Report on Preterm Birth* (Springer, 2012).

[4] Walani SR (2020). Global burden of preterm birth. Int. J. Gynaecol. Obstetr..

[5] World Health Organization (WHO). Preterm birth (2018, accessed 19 Feb 2018). https://www.who.int/news-room/fact-sheets/detail/preterm-birth.

[6] Chawanpaiboon S, Vogel JP, Moller A-B, Lumbiganon P, Petzold M, Hogan D (2019). Global, regional, and national estimates of levels of preterm birth in 2014: a systematic review and modelling analysis. Lancet Glob. Health.

[7] EVERY PREEMIE SCALE (USAID and PCI). Profile of preterm and low birth weight prevention and care. https://reliefweb.int/report/ethiopia/ethiopia-profile-preterm-and-low-birth-weight-prevention-and-care (2015).

[8] Muchie KF, Lakew AM, Teshome DF, Yenit MK, Sisay MM, Mekonnen FA (2020). Epidemiology of preterm birth in Ethiopia: systematic review and meta-analysis. BMC Pregn. Childbirth.

[9] Been JV, Millett C (2019). Reducing the global burden of preterm births. Lancet Glob. Health.

[10] World Health Organization (WHO). Preterm birth. https://www.who.int/news-room/fact-sheets/detail/preterm-birth (2018).

[11] Howe T-H, Sheu C-F, Wang T-N, Hsu Y-W (2014). Parenting stress in families with very low birth weight preterm infants in early infancy. Res. Dev. Disabil..

[12] Vogel JP, Chawanpaiboon S, Moller A-B, Watananirun K, Bonet M, Lumbiganon P (2018). The global epidemiology of preterm birth. Best Pract. Res. Clin. Obstet. Gynaecol..

[13] Liu L, Oza S, Hogan D, Chu Y, Perin J, Zhu J (2016). Global, regional, and national causes of under-5 mortality in 2000–15: An updated systematic analysis with implications for the Sustainable Development Goals. The Lancet..

[14] UN Inter-Agency Group for Child Mortality Estimation. Levels and trends in child mortality: Available on https://www.un.org/en/development/desa/population/publications/mortality/child-mortality-report-2017.asp. New York: United Nations Children’s Fund (2017).

[15] Wagura P, Wasunna A, Laving A, Wamalwa D, Nganga P (2018). Prevalence and factors associated with preterm birth at kenyatta national hospital. BMC Pregn. Childbirth.

[16] Derraik JGB, Lundgren M, Cutfield WS, Ahlsson F (2016). Maternal height and preterm birth: A study on 192,432 Swedish women. PLOS ONE.

[17] Delnord M, Blondel B, Zeitlin J (2015). What contributes to disparities in the preterm birth rate in European countries?. Curr. Opin. Obstetr. Gynecol..

[18] van den Broek NR, Jean-Baptiste R, Neilson JP (2014). Factors associated with preterm, early preterm and late preterm birth in Malawi. PLOS ONE..

[19] Sigalla GN, Mushi D, Meyrowitsch DW, Manongi R, Rogathi JJ, Gammeltoft T (2017). Intimate partner violence during pregnancy and its association with preterm birth and low birth weight in Tanzania: A prospective cohort study. PLOS ONE..

[20] Requejo J, Merialdi M, Althabe F, Keller M, Katz J, Menon R (2013). Born Too Soon: Care during pregnancy and childbirth to reduce preterm deliveries and improve health outcomes of the preterm baby. Reprod. Health.

[21] Muhumed II, Kebira JY, Mabalhin MO (2021). Preterm birth and associated factors among mothers who gave birth in Fafen Zone Public Hospitals, Somali Regional State, Eastern Ethiopia. Res. Rep. Neonatol..

[22] Richterman A, Raymonville M, Hossain A, Millien C, Joseph JP, Jerome G (2020). Food insecurity as a risk factor for preterm birth: A prospective facility-based cohort study in rural Haiti. BMJ Glob. Health.

[23] Eick SM, Meeker JD, Swartzendruber A, Rios-McConnell R, Brown P, Vélez-Vega C (2020). Relationships between psychosocial factors during pregnancy and preterm birth in Puerto Rico. PLOS ONE.

[24] Staneva A, Bogossian F, Pritchard M, Wittkowski A (2015). The effects of maternal depression, anxiety, and perceived stress during pregnancy on preterm birth: A systematic review. Women Birth.

[25] Moons KG, Altman DG, Vergouwe Y, Royston P (2009). Prognosis and prognostic research: application and impact of prognostic models in clinical practice. BMJ.

[26] Włodarczyk T, Płotka S, Szczepański T, Rokita P, Sochacki-Wójcicka N, Wójcicki J (2021). Machine learning methods for preterm birth prediction: A review. Electronics.

[27] Gomez R, Romero R, Medina L, Nien JK, Chaiworapongsa T, Carstens M (2005). Cervicovaginal fibronectin improves the prediction of preterm delivery based on sonographic cervical length in patients with preterm uterine contractions and intact membranes. Am. J. Obstetr. Gynecol..

[28] Saade GR, Boggess KA, Sullivan SA, Markenson GR, Iams JD, Coonrod DV (2016). Development and validation of a spontaneous preterm delivery predictor in asymptomatic women. Am. J. Obstetr. Gynecol..

[29] Ngo TT, Moufarrej MN, Rasmussen M-LH, Camunas-Soler J, Pan W, Okamoto J (2018). Noninvasive blood tests for fetal development predict gestational age and preterm delivery. Science.

[30] Katz J, Lee AC, Kozuki N, Lawn JE, Cousens S, Blencowe H (2013). Mortality risk in preterm and small-for-gestational-age infants in low-income and middle-income countries: A pooled country analysis. The Lancet.

[31] Hosny A, Aerts H (2019). Artificial intelligence for global health. Science.

[32] Huang D, Liu Z, Liu X, Bai Y, Wu M, Luo X (2021). Stress and metabolomics for prediction of spontaneous preterm birth: A prospective nested case-control study in a tertiary Hospital. Front. Pediatr..

[33] Feleke SF, Anteneh ZA, Wassie GT, Yalew AK, Dessie AM (2022). Developing and validating a risk prediction model for preterm birth at Felege Hiwot Comprehensive Specialized Hospital, North-West Ethiopia: A retrospective follow-up study. BMJ Open.

[34] Fente BM, Asaye MM, Tesema GA, Gudayu TW (2023). Development and validation of a prognosis risk score model for preterm birth among pregnant women who had antenatal care visit, Northwest, Ethiopia, retrospective follow-up study. BMC Pregn. Childbirth.

[35] Lee KJ, Yoo J, Kim Y-H, Kim SH, Kim SC, Kim YH (2020). The clinical usefulness of predictive models for preterm birth with potential benefits: A KOrean Preterm collaboratE Network (KOPEN) registry-linked data-based cohort study. Int. J. Med. Sci..

[36] Mailath-Pokorny M, Polterauer S, Kohl M, Kueronyai V, Worda K, Heinze G (2015). Individualized assessment of preterm birth risk using two modified prediction models. Eur. J. Obstetr. Gynecol. Reprod. Biol..

[37] Stock SJ, Horne M, Bruijn M, White H, Boyd KA, Heggie R (2021). Development and validation of a risk prediction model of preterm birth for women with preterm labour symptoms (the QUIDS study): A prospective cohort study and individual participant data meta-analysis. PLoS Med..

[38] Allouche M, Huissoud C, Guyard-Boileau B, Rouzier R, Parant O (2011). Development and validation of nomograms for predicting preterm delivery. Am. J. Obstetr. Gynecol..

[39] van de Mheen L, Schuit E, Lim AC, Porath MM, Papatsonis D, Erwich JJ (2014). Prediction of preterm birth in multiple pregnancies: development of a multivariable model including cervical length measurement at 16 to 21 weeks’ gestation. J. Obstetr. Gynaecol. Can..

[40] Zhang J, Zhan W, Lin Y, Yang D, Li L, Xue X (2021). Development and external validation of a nomogram for predicting preterm birth at< 32 weeks in twin pregnancy. Sci. Rep..

[41] Molla M. Butajira Butajira Rural Health Program (HDSS), Ethiopia. http://www.indepth-network.org/Profiles/butajira_hdss_2013.pdf (2013).

[42] Ververs M-T, Antierens A, Sackl A, Staderini N, Captier V (2013). Which anthropometric indicators identify a pregnant woman as acutely malnourished and predict adverse birth outcomes in the humanitarian context?. PLoS Curr..

[43] Sherin KM, Sinacore JM, Li XQ, Zitter RE, Shakil A (1998). HITS: A short domestic violence screening tool for use in a family practice setting. Fam. Med..

[44] Roberti JW, Harrington LN, Storch EA (2006). Further psychometric support for the 10-item version of the perceived stress scale. J. Coll. Counsel..

[45] World Health Organization. Haemoglobin concentrations for the diagnosis of anaemia and assessment of severity. World Health Organization (2011).

[46] Organization WH. WHO antenatal care randomized trial: manual for the implementation of the new model. In *World Health Organization. Report No.: 9241546298* (2002).

[47] R Core Team. R: A language and environment for statistical computing. In *R Foundation for Statistical Computing, Vienna, Austria* https://www.R-project.org/ (2021).

[48] Little RJ (1988). A test of missing completely at random for multivariate data with missing values. J. Am. Stat. Assoc..

[49] White IR, Royston P, Wood AM (2011). Multiple imputation using chained equations: issues and guidance for practice. Stat. Med..

[50] Tibshirani RJ, Efron B (1993). An introduction to the bootstrap. Monogr. Stat. Appl. Prob..

[51] Collins GS, Reitsma JB, Altman DG, Moons KG (2015). Transparent reporting of a multivariable prediction model for individual prognosis Or Diagnosis (TRIPOD): The TRIPOD Statement. Br. J. Surg..

[52] Youden WJ (1950). Index for rating diagnostic tests. Cancer.

[53] Schaaf JM, Ravelli AC, Mol BWJ, Abu-Hanna A (2012). Development of a prognostic model for predicting spontaneous singleton preterm birth. Eur. J. Obstetr. Gynecol. Reprod. Biol..

