# Supplementary material for: Development and validation of a simplified risk prediction model for preterm birth: a prospective cohort study in rural Ethiopia
Source: Sci Rep. 2024 Feb 28;14:4845. doi: 10.1038/s41598-024-55627-z (PMC10901814; doi:10.1038/s41598-024-55627-z)
Supplement: Supplementary file 1 — Supplementary Tables. [file 41598_2024_55627_MOESM1_ESM.docx]

**Supplementary Table 1: Sensitivity analysis for comparison of the complete case and multiple imputations of the individual predictors of preterm birth**

| **Predictors**^¶^ | **Complete case analysis** | | | **Multiple imputations** | | |
| --- | --- | --- | --- | --- | --- | --- |
|  | **coefficients** | **95% CI** | **P-value** | **coefficients** | **95% CI** | **P-value** |
| Age less than 20 | -0.74 | -1.80,0.32 | 0.170 | -0.73 | -1.79, 0.33 | 0.179 |
| Age ≥35 | 0.06 | -0.96,1.08 | 0.907 | 0.08 | -0.94, 1.10 | 0.874 |
| No formal education | -0.46 | -1.17,0.25 | 0.226 | -0.46 | -1.17, 0.25 | 0.203 |
| Primary | -0.43 | -1.14,0.28 | 0.226 | -0.43 | -1.14, 0.28 | 0.223 |
| MUAC <23 cm | 0.51 | -0.04,1.06 | 0.065 | 0.51 | -0.04, 1.06 | 0.066 |
| Primigravida | 0.42 | -0.12,0.96 | 0.131 | 0.48 | -0.01, 0.97 | 0.056 |
| Had a history of abortion | 1.24 | 0.38,2.10 | 0.001 | 1.00 | 0.37, 1.63 | 0.003 |
| Had a history of preterm birth | 0.74 | -0.97, 2.45 | 0.396 | 0.46 | -0.46, 1.38 | 0.338 |
| Anaemic | 1.08 | 0.32,1.84 | 0.006 | 1.04 | 0.28, 1.80 | 0.008 |
| Positive IPV | 0.85 | 0.20,1.50 | 0.011 | 0.85 | 0.20, 1.50 | 0.011 |
| History of contraceptive use | -0.10 | -0.63,0.43 | 0.719 | -0.10 | -0.63, 0.43 | 0.715 |
| Unplanned pregnancy | 0.24 | -0.33,0.81 | 0.411 | 0.064 | -0.52, 0.65 | 0.829 |
| History of ANC for current pregnancy | 0.73 | 0.04,1.42 | 0.036 | 0.72 | 0.03, 1.41 | 0.037 |
| History of ANC for last pregnancy | 0.08 | -0.57,0.73 | 0.818 | 0.26 | -0.47, 0.99 | 0.482 |
| Deworming | -0.38 | -1.36,0.60 | 0.45 | -0.39 | -1.37, 0.59 | 0.443 |
| ^#^Comorbidity | 0.98 | 0.25,1.71 | 0.008 | 0.94 | 0.21, 1.67 | 0.011 |
| Substance use | -0.38 | -1.12,0.36 | 0.322 | -0.38 | -1.12, 0.36 | 0.315 |
| Moderate maternal stress | 0.60 | -0.01,1.21 | 0.051 | 0.60 | -0.01, 1.21 | 0.052 |
| High maternal stress | 1.41 | 0.25,2.57 | 0.016 | 1.406 | 0.26,2.55 | 0.06 |
| ^¶^Predictors of the preterm birth; ^#^Comorbidity included medical conditions, either cardiac disease or diabetes disease, chronic hypertension or HIV infection, malaria, or renal disease; MUAC: Mid-Upper Arm Circumference; ANC: Antenatal Care, IPV: Intimate Partner Violence | | | | | | |

**Supplementary Table 2: TRIPOD Checklist: Development and validation of a simplified risk prediction model for preterm birth**

| **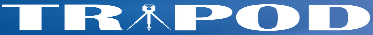TRI TRIPOD Checklist: Development and validation of a simplified risk prediction model for preterm birth** | | | | |
| --- | --- | --- | --- | --- |
| **Section/Topic** |  |  | **Checklist Item** | **Page** |
| **Title and abstract** | | | | |
| Title | 1 | D;V | Identify the study as developing and/or validating a multivariable prediction model, the target population, and the outcome to be predicted. | 1 |
| Abstract | 2 | D;V | Provide a summary of objectives, study design, setting, participants, sample size, predictors, outcome, statistical analysis, results, and conclusions. | 2 |
| **Introduction** | | | | |
| Background and objectives | 3a | D;V | Explain the medical context (including whether diagnostic or prognostic) and rationale for developing or validating the multivariable prediction model, including references to existing models. | 3 |
|  | 3b | D;V | Specify the objectives, including whether the study describes the development or validation of the model or both. | 3&4 |
| **Methods** | | | | |
| Source of data | 4a | D;V | Describe the study design or source of data (e.g., randomized trial, cohort, or registry data), separately for the development and validation of data sets, if applicable. | 3 |
|  | 4b | D;V | Specify the key study dates, including the start of accrual; end of accrual; and, if applicable, end of follow-up. | 3 |
| Participants | 5a | D;V | Specify key elements of the study setting (e.g., primary care, secondary care, general population) including number and location of centres. | 3&4 |
|  | 5b | D;V | Describe eligibility criteria for participants. | 4 |
|  | 5c | D;V | Give details of treatments received, if relevant. | NA |
| Outcome | 6a | D;V | Clearly define the outcome that is predicted by the prediction model, including how and when assessed. | 4&5 |
|  | 6b | D;V | Report any actions to blind assessment of the outcome to be predicted. | NA |
| Predictors | 7a | D;V | Clearly define all predictors used in developing or validating the multivariable prediction model, including how and when they were measured. | 5&6 |
|  | 7b | D;V | Report any actions to blind assessment of predictors for the outcome and other predictors. | NA |
| Sample size | 8 | D;V | Explain how the study size was arrived at. | NA |
| Missing data | 9 | D;V | Describe how missing data were handled (e.g., complete-case analysis, single imputation, multiple imputations) with details of any imputation method. | 6 |
| Statistical analysis methods | 10a | D | Describe how predictors were handled in the analyses. | 6&7 |
|  | 10b | D | Specify the type of model, all model-building procedures (including any predictor selection), and the method for internal validation. | 6&7 |
|  | 10c | V | For validation, describe how the predictions were calculated. | 7 |
|  | 10d | D;V | Specify all measures used to assess model performance and, if relevant, to compare multiple models. | 6&7 |
|  | 10e | V | Describe any model updating (e.g., recalibration) arising from the validation, if done. | 6&7 |
| Risk groups | 11 | D;V | Provide details on how risk groups were created, if done. | 6&7 |
| Development vs. validation | 12 | V | For validation, identify any differences from the development data in setting, eligibility criteria, outcome, and predictors. | NA |
| **Results** | | | | |
| Participants | 13a | D;V | Describe the flow of participants through the study, including the number of participants with and without the outcome and, if applicable, a summary of the follow-up time. A diagram may be helpful. | 7&8 |
|  | 13b | D;V | Describe the characteristics of the participants (basic demographics, clinical features, available predictors), including the number of participants with missing data for predictors and outcome. | 7&8 |
|  | 13c | V | For validation, show a comparison with the development data of the distribution of important variables (demographics, predictors and outcome). | 9&10 |
| Model development | 14a | D | Specify the number of participants and outcome events in each analysis. | 9&10 |
|  | 14b | D | If done, report the unadjusted association between each candidate predictor and outcome. | 9&10 |
| Model specification | 15a | D | Present the full prediction model to allow predictions for individuals (i.e., all regression coefficients, and model intercept or baseline survival at a given time point). | 9,10&11 |
|  | 15b | D | Explain how to the use the prediction model. | 9&10 |
| Model performance | 16 | D;V | Report performance measures (with CIs) for the prediction model. | 10&11 |
| Model-updating | 17 | V | If done, report the results from any model updating (i.e., model specification, model performance). | 10-13 |
| **Discussion** | | | | |
| Limitations | 18 | D;V | Discuss any limitations of the study (such as a nonrepresentative sample, few events per predictor, or missing data). | 15 |
| Interpretation | 19a | V | For validation, discuss the results with reference to performance in the development data, and any other validation data. | 14 |
|  | 19b | D;V | Give an overall interpretation of the results, considering objectives, limitations, results from similar studies, and other relevant evidence. | 14&15 |
| Implications | 20 | D;V | Discuss the potential clinical use of the model and its implications for future research. | 15 |
| **Other information** | | | | |
| Supplementary information | 21 | D;V | Provide information about the availability of supplementary resources, such as study protocol, Web calculator, and data sets. | 16 |
| Funding | 22 | D;V | Give the source of funding and the role of the funders for the present study. | 16 |

*Items relevant only to the development of a prediction model are denoted by D, items relating solely to the validation of a prediction model are denoted by V, and items relating to both are denoted by D;V. We recommend using the TRIPOD Checklist in conjunction with the TRIPOD Explanation and Elaboration document.
